# An analysis of the entrainment effect of dry debris avalanches on loose bed materials

**DOI:** 10.1186/s40064-016-3272-4

**Published:** 2016-09-20

**Authors:** Peng-yuan Lu, Xing-guo Yang, Fu-gang Xu, Tian-xing Hou, Jia-wen Zhou

**Affiliations:** 1 State Key Laboratory of Hydraulics and Mountain River Engineering, Sichuan University, Chengdu, 610065 People’s Republic of China; 2 College of Water Resource and Hydropower, Sichuan University, Chengdu, 610065 People’s Republic of China

**Keywords:** Debris avalanche, Physical modeling test, Impact erosion, Basal abrasion, Entrainment mechanism

## Abstract

Substrate entrainment can greatly influence the mass movement process of a debris avalanche because it can enlarge the landslide volume and change the motion characteristics of the sliding masses. To study the interaction between debris avalanches and erodible substrate, physical modeling experiments varying in the mass of granular flow and substrate thickness were performed. The experimental results show that both the entrained materials and the maximum erosion depth are increased with increasing mass of the debris avalanche and decreasing substrate thickness. During the experiment, several tests were recorded using a high-speed digital camera with a frequency of 500 frames per second, so that the process of entrainment could be clearly observed. Combined with the experiment result and results of previous studies from predecessors, the entrainment mechanism during debris avalanches are analyzed and discussed. The entrainment effect of the sliding masses on the loose bed materials include basal abrasion and impact erosion of the avalanche front, the latter of which can contribute to the former by failing or yielding the erodible bed.

## Background

In recent years, the occurrence frequency of catastrophic landslides has significantly increased because of human activities and changes in global climate (e.g., rising temperature and heavy rainfall), causing a tragic loss of lives and properties (Crosta et al. 2009; He et al. 2010; Monjez et al. 2011; Zhou et al. 2013; Singh et al. 2014; Umrao et al. 2015). However, most catastrophic landslides are triggered by strong earthquakes (Ding et al. 2012; Wu et al. 2012; Zhou et al. 2016a), e.g., the 2008 Wenchuan earthquake directly caused more than 15,000 geohazards in the form of landslides, rockfalls and debris flows, which resulted in approximately 20,000 deaths (Yin et al. 2009; Xing et al. 2015). The 2010 Haiti earthquake triggered tens of thousands of landslides, killed more than 200,000 people and caused approximately $8 billion in damages (Calais et al. 2010; Xu et al. 2012). Field investigations indicate that the magnitude of debris avalanches can rarely be determined by the volume of the initiating landslide, which may be small, as they can increase by several times during the moving process (Hungr et al. 2005). Landslide paths are often covered by surficial materials, including colluvium, glacial till, residual soil, alluvium and organics, which may be loose and saturated (McDougall and Hungr 2005). During the motion process of debris avalanches, rapid sliding masses will interact with the bed materials along the traveling paths and then entrain them (Legros 2002; Dufresne 2012). The entrainment of bed materials can increase the volume of the landslide by several times, alter avalanche characteristics because of the involvement of substrate materials, and enhance avalanche mobility (Hungr and Evans 2004; McDougall and Hungr 2005).

Because the entrainment effect is critical to the motion process of debris avalanches, considerable attention has been given to this topic over a long time span, and many cases involving this phenomenon have been well documented. For example, the 2000 Yigong landslide, with an initial volume of approximately 1 × 10^8^ m^3^, increased its volume to more than 3 × 10^8^ m^3^ because of the entrainment of a large amount of bed materials (colluvium with snow and ice) along the moving path (Shang et al. 2003). Zhou et al. (2016a) considered that there are three types of entrainment modes for high-speed landslides, namely, impact, scouring and erosion, and that high motion speed and water flow were primary factors for the intense entrainment of the Yigong landslide. The 1999 Nomash River landslide, with an initial volume of 300,000 m^3^, entrained approximately 360,000 m^3^ of materials, with an entrainment ratio of 0.96 (McDougall and Hungr 2005). The Val Pola rock avalanche entrained approximately 8 × 10^6^ m^3^ of bed materials, which was approximately 20 % of the initial volume (Crosta et al. 2004). Crosta (1994) reported a rock slide/avalanche with an original volume of 0.15 × 10^6^ m^3^ entrained approximately 0.25 × 10^6^ m^3^ of materials while moving along a 35° inclined slope covered with deposit. Based on a large number of reported landslide cases, the entrainment effect is a common phenomenon in many large-scale landslides.

Previous studies on the entrainment effect have included experiments and numerical simulations with variable emphases or considering different factors. For example, Zhou et al. (2016a) studied the effect of the weight ratio of a debris avalanche between substrate and water on entrainment, indicating that landslides entrain bed materials via impact failure, scouring and erosion, and water plays a key role in this process. Dufresne (2012), through a small-scale laboratory test, found that increasing basal boundary roughness of the substrate increases the stability of the chain force and results in a shorter runout distance and that entrainment mechanisms include ploughing, deformation waves, fluidization and basal abrasion. Mangeney et al. (2010) conducted research on erosion and mobility in a granular collapse, drawing a conclusion that the erosion efficiency strongly increases with increasing slope. Furthermore, different numerical methods are conducted to study the entrainment effect of landslides on bed materials under different conditions (McDougall and Hungr 2005; McDougall et al. 2006; Mangeney et al. 2007; Crosta et al. 2015), and the discrete element method is widely used for simulation of the entrainment effect during mass movement processes. However, the entrainment effect of landslides on bed materials is a complicated problem, which is not only related to the volume and velocity of the flows but also related with the properties of the substrate materials (e.g., density, water content, grain graduation and others). In this paper, according to the entrainment effect of debris avalanches on loose bed materials, a flume is designed to simulate a debris avalanche on a slope, and several laboratory tests under different test conditions are performed. During the experimental process, a high-speed camera was used to record the mass movement process. Mass movement, entrainment and deposition of the sliding masses are analyzed. Combined with previous research results and the physical modeling test results, a new understanding on the entrainment mechanism of debris avalanches on loose bed materials is presented.

## Methods

Many studies have indicated that substrate entrainment can alter the mobility of debris avalanches and greatly enlarge its volume, which may cause more severe catastrophes (Hungr and Evans 2004; Mangeney et al. 2010). In this paper, a flume is designed to study the entrainment effect of dry debris avalanches on loose bed materials by using friction model. The detail information of the flume and the use of materials, test conditions and experimental processes are briefly introduced in this section.

### Experiment setup

**Fig. 1:**
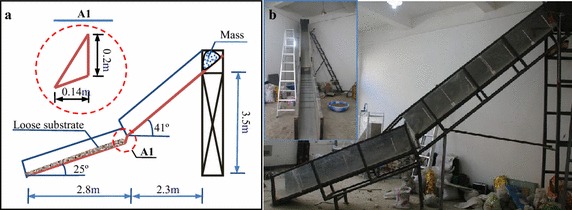
Physical modeling test apparatus for the debris avalanche: **a** structural size design and **b** site photos of the flume

The total length and height of the experimental flume are 5.1 and 3.5 m, respectively, with width and depth of 40 and 50 cm, respectively (as shown Fig. 1a). The experimental flume consists of four parts: topper hopper, movement section, connection section and accumulation section. The topper hopper is a material box to simulate the initiation of the debris avalanche, with a length of 70 cm, width of 40 cm, height of 70 cm, capacity of 154 L, and a floor with an inclination of 24°. To accelerate the debris avalanche to a relatively large velocity, the movement section is set at a steep slope of 41°, with a length of 3.0 m. The length of the accumulation section is 2.9 m, with a slope of 25°, and the loose bed materials are accumulated in this section.

As shown in Fig. 1b, the terrain of the debris avalanche path changes everywhere, which inevitably causes the projection of the flow and the impact with the substrate. Therefore, an elevation difference between the moving and accumulation section was designed, connecting with a board, with a length of 28 cm and inclination of 60°. To observe the entrainment process, one side of the flume is made of transparent organic glass, and the other side and the base are made of organic plastics. Furthermore, several bars were glued on the flume surface of the accumulation section to increase the frictional resistance, which can avoid shear failure at this contact surface between the flume and loose bed materials.

### Experiment design

**Fig. 2:**
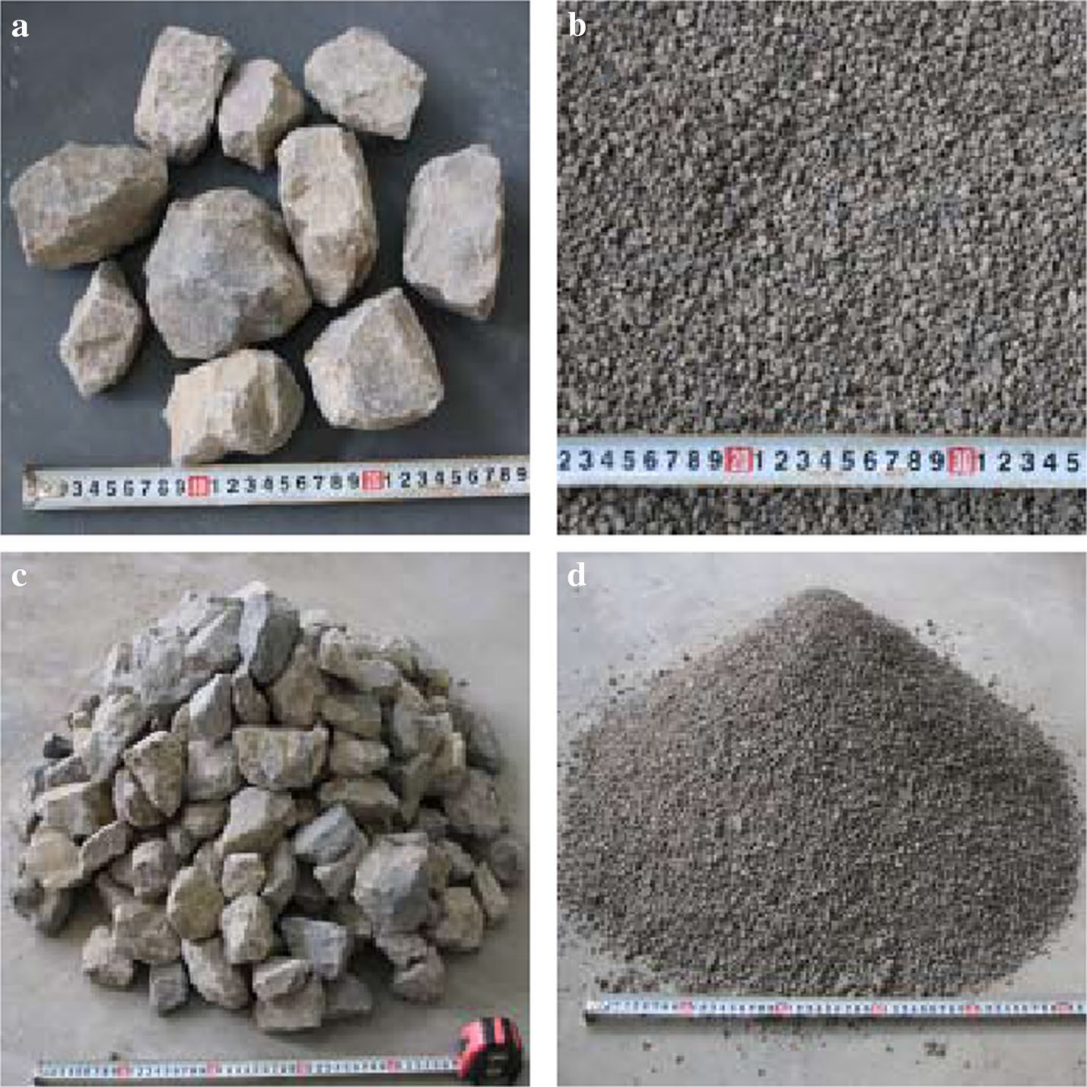
Fragment materials used for the laboratory tests: **a** 40–80 mm; **b** 2–5 mm; **c** repose angle of the debris avalanche materials and **d** repose angle of the substrate materials

The materials used in the laboratory tests are collected from a landslide site at Qingping, Sichuan, Southwest China, and include debris materials generated from the catastrophic landslides triggered by the 2008 Wenchuan earthquake. After sieving the raw materials, two groups of materials with different particle sizes of 2–5 and 40–80 mm were used in our laboratory tests (Fig. 2a, b). A group with a size of 40–80 mm is used to simulate the debris avalanche, whereas the other (2–5 mm) is used to simulate the loose bed materials. The repose angles of the fine-grained and coarse materials are approximately 31°–33° (Fig. 2c) and 28°–30° (Fig. 2d), respectively.

The physical modeling test used to study the entrainment effect of dry debris avalanches on loose bed materials is a kind of friction model. During the mass movement process of the debris avalanches, the energy of sliding masses is transmitted to the substrate materials and then entrains them. The energy of the avalanche originates from the potential energy of the source materials, which means that different debris mass or volumes result in different amounts of energy and thus have different entrainment effects on bed materials. According to previous research, the substrate thickness is of great influence to the entrainment of bed materials. During the experimental process, based on these two factors in the entrainment process, 12 tests are conducted under different conditions (Table 1).

**Table 1:** Experimental design and entrainment effect test results

No.	Mass of debris avalanche (kg)	Maximum thickness of bed materials (cm)	Maximum entrainment depth (cm)
1	48	13.0	7.6
		16.0	6.9
		19.0	6.0
2	60	13.0	9.6
		16.0	8.9
		19.0	7.5
3	80	13.0	12.3
		16.0	11.0
		19.0	8.1
4	100	13.0	12.3
		16.0	11.5
		19.0	8.6

**Fig. 3:**
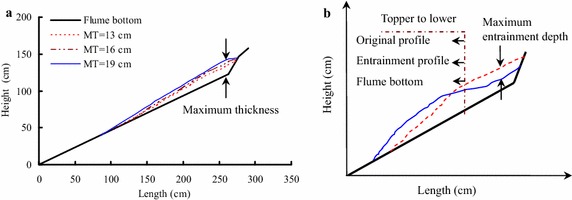
**a** Original profile of the loose bed materials accumulated on the flume (MT is the maximum thickness of the bed materials); **b** the definition of the maximum entrainment depth for the bed materials

The definition of the maximum thickness of bed materials is shown in Fig. 3a, and the definition of maximum entrainment depth is shown in Fig. 3b

Three different bed material thicknesses are used for the laboratory tests. Figure 3a shows the original profile of the loose bed materials accumulated on the flume.

As shown in Fig. 3a, most of the bed materials are accumulated at the front of the lower part of the flume, and three maximum thickness conditions of the bed materials are used here, which include 13, 16 and 19. Furthermore, the accumulated shape of the bed materials in the flume is an irregular triangle, and the accumulation thickness is decreased with decreasing height. Once the debris avalanche is initiated, the bed materials will be entrained by the sliding masses, and the final accumulation shape of the bed materials will be changed. As shown in Fig. 3b, the entrainment profile refers to the bed materials accumulated in the flume, and the entrainment depth is the difference between the original profile and the entrainment profile of the bed materials (where the maximum entrainment depth is the corresponding maximum value).

### Experimental process

Before the start of the laboratory tests, regular squared grids were drawn on the transparent side of the flume (the original profile of the bed materials is measured previously), and then the whole mass movement process and final deposition shape can be observed using a high-speed camera, a digital camera and a Single Lens Reflex (SLR) camera. The fine bed materials were paved in different thicknesses on the accumulation section, and the thickness of the erodible bed in different locations were measured. A determined mass of the coarse materials was taken into the hopper to simulate the sliding masses of debris avalanche. During the experimental process, a MotionPro-Y3-S1 type high-speed camera with a determined frequency (500 frames/s) was used to record the entrainment process of the sliding masses on the bed materials. Additionally, a digital camera in front of the flume was used to observe the moving process of the whole sliding masses. Once the gate of the hopper was opened, the debris avalanche was initiated and the masses would transport in the flume, and then entrained the bed materials. After the mass movement process is finished, the final profile (entrainment profile) of the bed materials is measured again, and several photos are taken from different positions. Some key phenomena and parameters are indirectly determined using the videos and photos.

## Experimental results

In this section, experimental results related with the mass movement and entrainment processes are first analyzed, and then comparative analyses of the deposition profile of bed materials under different conditions are conducted. Mass of debris avalanche and thickness of bed materials are the key factors affecting the entrainment process of debris avalanche discussed here.

### Mass movement

**Fig. 4:**
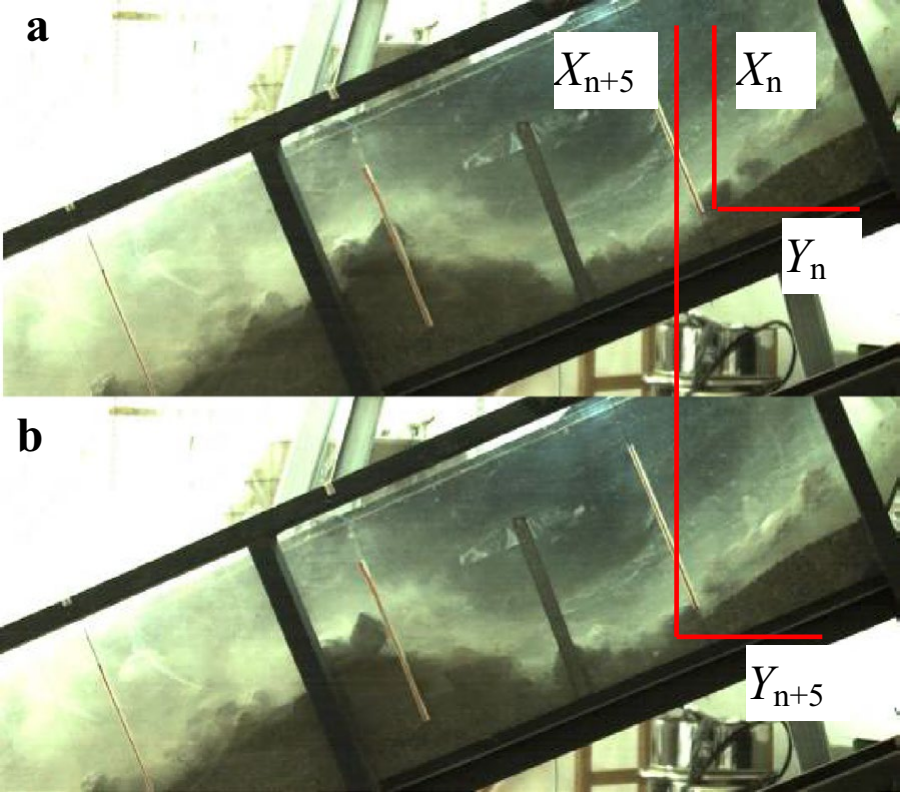
Moving distance of a typical particle measured by Digimizer software, an example of frame 810 (**a**) and frame 815 (**b**), with a time step of 5/500 s

Mass movement characteristics are important parameters for the entrainment of bed materials, along with the structural and mechanical properties of the bed materials. The frequency of the high-speed digital camera used in the laboratory test is very high (500 frames/s) that the velocity can be measured manually from the monitoring videos. Furthermore, the length of time between two or several sequential frames is very small, so the average velocity can be taken as an instantaneous parameter of the moving particles. According to the high-speed camera frames, the *X* (horizontal direction) and *Y* (vertical direction) coordinates in any position of the image can be obtained using Digimizer free image analysis software, Version 4.3.0 (MedCalc Software, Mariakerke, Belgium), so that the moving distance components of typical particles between two different frames can be calculated. Figure 4 shows an example for the moving distance of one particle in 6 frames.

As shown in Fig. 4, the moving distance of one sliding particle in the *X* and *Y* direction can be easily determined from the segmented images. Here, the moving distance components of the typical particles are determined by the images of every 6 frames, which can be calculated as follows (Barbolini et al. 2005a):

1 $$\begin{aligned} &\Delta X = X_{n + 5} - X_{n} \\ &\Delta Y = Y_{n + 5} - Y_{n} \\ \end{aligned}$$

where Δ*X* and Δ*Y* are the moving distances of 6 frames in the horizontal direction and vertical direction, respectively; *X*_*n*_ and *Y*_*n*_ are the coordinates in the *X* and *Y* directions in frame *n*, respectively; *X*_*n*+5_ and *Y*_*n*+5_ are the coordinates in the *X* and *Y* directions in frame *n* + 5, respectively.

According to the moving distance components of one typical particle, the horizontal velocity (*V*_*xn*_) and vertical velocity (*V*_*yn*_) can be determined as follows:

2 $$\begin{aligned} & V_{xn} = \frac{{\Delta X}}{{\left( {{1 \mathord{\left/ {\vphantom {1 {100}}} \right. \kern-0pt} {100}}} \right)}} \\ & V_{yn} = \frac{{\Delta Y}}{{\left( {{1 \mathord{\left/ {\vphantom {1 {100}}} \right. \kern-0pt} {100}}} \right)}} \\ \end{aligned}$$

Therefore, the overall velocity of the typical particle can be computed as follows:

3 $$V_{n} = \sqrt {V_{xn}^{2} + V_{yn}^{2} }$$

**Fig. 5:**
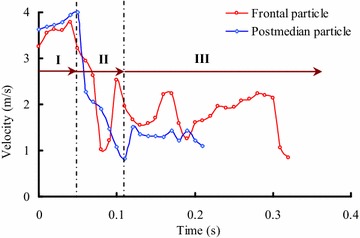
Evolution of velocity of typical particles in sliding masses (when the mass of debris avalanche is 100 kg and maximum thickness of the bed materials is 13 cm)

During the mass movement process of sliding masses, the motion velocity of every particle is constantly changing because of energy conversion and dissipation, interaction between the upper sliding masses and bed materials or different particles. Figure 5 shows the evolution process for the velocities of two particles in the front and postmedian of the sliding masses (when the debris mass is 100 kg and maximum thickness of the bed materials is 13 cm). As shown in Fig. 5, the origin coordinates (time is 0) indicate the time at which the particles begin to enter the field of vision of the high-speed camera. The velocity of the debris increases for a little while because it experiences a short acceleration process before impacting the bed materials. The debris leaves the field of vision of the high-speed camera at the end point in Fig. 5, and it will continue to move forward until it stops.

There are three stages for the upper motion debris: stage I is when the motion velocity continues to increase, although the debris has not yet been in contact with the substrate materials. As shown in Fig. 5, the motion velocities of particles both in front and postmedian are slightly increased after they leave the movement section. Stage II is when the motion velocity rapidly decelerates, and strong interactions occur between the motion debris and substrate materials (impacting, scouring and erosion cause great energy dissipation for the motion debris). The velocity sharply decreases when the sliding masses encounter the bed materials accumulated in the flume, activating the bed materials through the transmission of kinetic energy. The frontal particle will bound up and then undergo a process of deceleration and acceleration in sequence. The process of impact with the substrate and bouncing off the bed can last for several cycles, but the fragment will slide along the bed with decreasing velocity. Stage III is the continued motion process, with a gradual decrease in the overall velocity as the bed materials mix into the upper motion debris. The particles in the postmedian section bounce off the bed the first time it encounters the substrate, and then move down along the surface of the bed, decelerating until they stop. During the deceleration process, part of the consumed energy will be transmitted to the static substrate materials which then are entrained. Furthermore, the velocity fluctuates when the particles move forward because of the collision of the following particles.

### Entrainment process

**Fig. 6:**
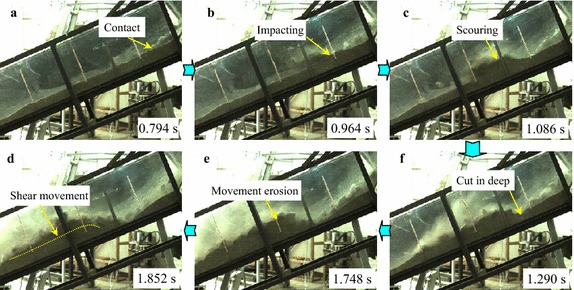
Entrainment process of the debris avalanche on the bed materials: **a** the debris avalanche encounters erodible substrate; **b** the bed material is sprayed by the high-speed debris because of the impact; **c**, **d** the bed materials are scoured by the motion of the debris and gradually cut in deep; **e** shallow erosion occurs and compression ridge forms because of collision and compaction; **f** a thin layer of substrate materials is activated to move forward under the shear effect of upper motion debris

During the experiment, high-speed camera with high frequency is used to record the entrainment process of a debris avalanche on the bed materials, and the entrainment process can be clearly observed. Figure 6 shows the typical entrainment process of the sliding masses on the loose bed materials.

As shown in Fig. 6b, once the high-speed sliding masses encounter the bed materials, an intense impact occurs causing the bed materials to spray forward with a high velocity. After the impact with the substrate, some debris materials bound upward and make additional contact with the bed, which causes secondary collisions. With the bed materials constantly impacted by the upper sliding masses, the bed materials are scoured by the debris and gradually causing deep cuts (Fig. 6c, d). Hence, the subsequent mass will not cause further spray of the bed materials, but instead compresses them and pushes the material forward. During the impact and scouring of the bed materials at the impact area, compression ridge forms in front of this position because of collision and compression of the debris avalanche on the frontal static substrate materials (Marr et al. 2001). Furthermore, a thin layer on the top of the substrate is activated to move under the drag of the debris avalanche because the bed materials are loosely piled and have low resistance strength. According to the videos, the velocity of this layer is slightly slower than the debris avalanche, but it moves synchronously after being accelerated. The thickness of this layer becomes thinner along the accumulation section. In the moving process of the entrained materials, some of the materials are involved in the debris avalanche and move forward together with the debris avalanche. The debris loses considerable energy during the collision with the substrate. Therefore, its velocity decreases substantially at the impact zone, which hinders the following mass from moving forward. As a result, a majority of the debris materials are deposited near the impact area. In the process of our experiment, injection of the debris into the substrate was also observed.

### Deposition profile of bed materials

**Fig. 7:**
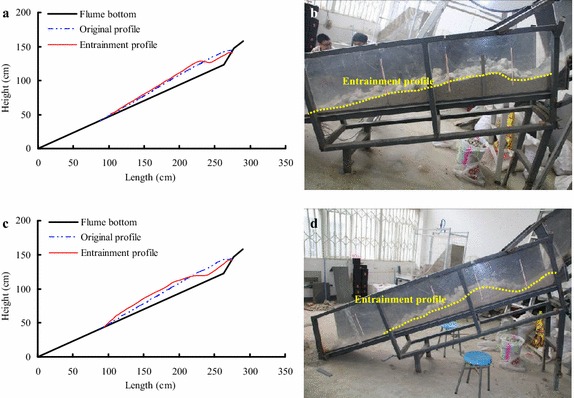
Entrainment effect of bed materials under different mass conditions (the maximum thickness of bed materials is 19 cm): **a** and **b** are the entrainment profiles under a mass of 48 kg; **c** and **d** are the entrainment profile under a mass of 80 kg

According to the thickness of the bed materials measured at different locations before and after the tests, the original shape and final deposition profile of the substrate in different conditions can be determined, respectively. Before releasing the slide materials, the substrate was piled on the accumulation section, with a relatively larger thickness on the top of this section and thinning along the flume. When the test begins, the high-speed debris avalanche impacts the bed materials, causing entrainment. The different debris mass suggest that the debris avalanche has different amounts of energy, which thus cause different amounts of entrained materials and erosion depths (Fig. 7).

**Fig. 8:**
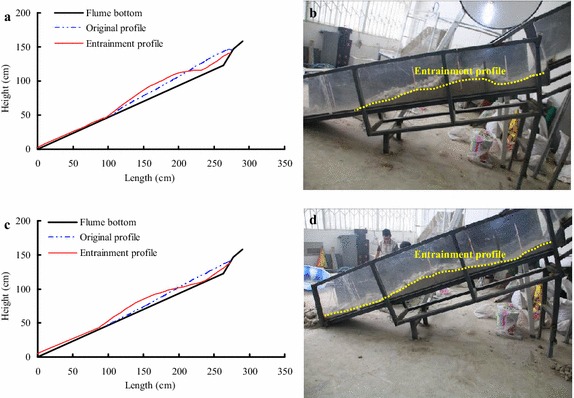
Deposition profile of substrate under conditions of different maximum substrate thicknesses (the mass is 100 kg): **a** the maximum thickness of the bed material is 19 cm; **b** the maximum thickness of the bed material is 13 cm

As shown in Fig. 7, the erosion depth increases with debris mass growth. For example, when the mass of debris avalanche increases from 48 to 80 kg, and the maximum thickness of the bed materials is 19 cm, the maximum erosion depth increases from 6.0 to 8.1 cm (as shown in Table 1). However, if the debris mass is larger than 80 kg, the maximum erosion depth depends on the substrate thickness in the erosion area. The front impact and scouring effect and the postmedian motion erosion effect of the debris avalanche on the bed materials are all increased with increasing sliding debris mass. Figure 8 shows the deposition profile of substrate under the conditions of different maximum substrate thicknesses (the mass of debris avalanche is 100 kg).

**Fig. 9:**
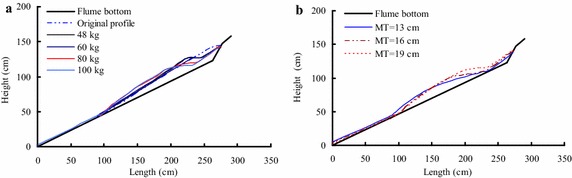
Comparative analyses for the final entrainment profile of bed materials under different conditions: **a** effect of the mass of debris avalanche (where the maximum thickness of the bed material is 19 cm) and **b** effect of bed material thickness (where the mass of debris avalanche is 100 kg)

As shown in Fig. 8, the experimental results indicate that the maximum erosion depth increases with decreasing substrate thickness. For instance, by increasing the maximum thickness of the bed materials from 13 to 19 cm and maintaining the mass of debris avalanche at 100 kg, the erosion depth decreases substantially from 12.3 to 8.6 cm (Table 1). As shown in Table 1, the experimental results indicate that the maximum erosion depth does not decrease linearly with the growth of the maximum substrate thickness. Figure 9 shows the comparative analyses for the final entrainment profile of bed materials under different conditions.

As shown in Fig. 9a and Table 1, the entrainment effect increases with increasing debris mass, this is because a growing mass of the debris avalanche means it contains greater motion energy. Besides, increasing mass also can lead to longer lasting of the erosion process, which can cause more serious impact, scouring and erosion of the bed materials. As shown in Fig. 9b and Table 1, the entrainment effect decreases with the increasing maximum thickness of bed materials, which indicates that greater thickness of the bed material resulted in more shear resistance to prevent the occurrence of shear failure. In a real large-scale debris avalanche, although the sliding masses have substantial kinetic energy, only a small depth of bed materials is entrained by the debris avalanche. The entrainment effect generally occurs on the shallow slope, and rarely occurs in the deep part of the slope.

## Discussion

### Scale effect

The scale of the modeling test in our experiment is much smaller than those characteristic of the natural debris avalanche. There have some implicit relationship between the small scale of physical modeling test and the large scale of actual debris avalanche, which is associated with the scale ratio in different aspects. A scaled model is a reduced representation of reality, and it needs to be geometrically and dynamically similar to field events (Hubbert 1937; Ramberg 1981). Thus, it is very important to study the scale effect and the relevance between our results and corresponding quantity of the field debris avalanche.

Regarding to the geometrical scaling of the model, the length scale ratio (*λ*_L_) which is defined as the ratio of the length in the model to the corresponding length in the prototype is used herein. By using of the length scale ratio (such as 1/50), some other geometrical variables like the landslide volume and entrainment depth of the scaled-up debris avalanche can be calculated. Actually, same scaling ratio should be chosen for the particle size of the debris materials and substrate materials to fulfill the geometrical scaling. However, if the same scale ratio is selected size of experimental materials, the particle size of substrate materials will be too fine which can lead to the fact that the resistance of the bed materials will be too large to be entrained. Besides, it is not conducive to observe the entrainment process because of the aroused great dust. In fact, it is impossible to exactly represent the prototype grains at small-scale modeling tests, where larger size for experimental materials is often chosen for some certain purposes (Davies and McSaveney 1999). Thus a larger scale ratio for the particle size of the experiment materials (*λ*_*D*_ = 1/20) can be selected in the modeling tests. Meanwhile, to maintain the same scale ratio of debris materials and substrate materials, larger size of the debris materials was chosen, which are which are 0.04–0.08 and 0.002–0.005 m for the debris avalanche and substrate materials.

In addition to geometrical similarity, dynamically similarity should be fulfilled between the flow in the experiment and field avalanche. However, it is difficult to completely attain at a small-scale because of high stress, grain comminution, and other dynamic process acting in rock and debris avalanche (Iverson et al. 2010; Dufresne 2012). To achieve a rough dynamic similarity, the Froude number, defined as the ratio of inertial to potential energy (Eq. 4), is an important dimensionless number that characterizes the dynamic of the flow and is required a similitude (Gleirscher and Fischer 2014).

4 $$F_{\text{r}} = \frac{U}{{\sqrt {gH} }}$$

where *F*_*r*_ denotes the Froude number; *U* denotes the flow velocity; *g* denotes gravity acceleration; *H* denotes flow thickness.

Froude scaling has been successfully applied in the experiments of debris flow and granular flow (Kaitna et al. 2006; Paola et al. 2009; Schneider et al. 2011). Under the condition of dynamically and geometrical similitude, the dynamic variables of the natural debris avalanche can be scaled up roughly based on the length scale ratio and height scale ratio. For simplicity, the values of density of the debris avalanche and substrate as well as the gravity of the model are assumed the same as the prototype. Under this condition, the corresponding variables of the natural debris avalanche which fulfilled the above similarity conditions can be scaled up. For example, in our experiment, the granular flow with volume and particle size of about 0.027–0.030 m^3^ and 0.04–0.08 m respectively can reach a velocity of 1.5–3.5 m/s and cause a entrainment depth of about 0.075 m when the initial substrate thickness is 0.19 m. Assuming that *λ*_L_ = 1/50, the volume and grain size of translated debris avalanche is (3.33–3.75) × 10^3^ and 0.8–1.6 m respectively. When it moves down the path with the with total height and length of 175 and 255 m which is similar to the model, its velocity can reach about 10.6–24.7 m/s and it can entrain bed materials with a maximum thickness about 3.75 m when the initial substrate thickness is 9.5 m (Table 2). Although these values do not apply to any specifics landslide–debris avalanche, they do correspond to the correct order of magnitude for a variety of field case (Marr et al. 2001).

**Table 2:** Scaling parameters and dimensionless scaling criteria (an example when the debris mass is 60 kg and the maximum substrate thickness is 19 cm)

Variables	Model value	Scale ratio	Prototype value
*ρ*_d_ (g/cm^3^)	2.00–2.25	1/1	2.00–2.25
*θ* (°)	41, 25	1/1	41, 25
*g* (m/s^2^)	9.8	1/1	9.8
*M*_d_ (kg)	60	*λ*_*M*_ = (*λ*_*L*_)^3^ = (1/50)^3^	7.5 × 10^6^
*V*_d_ (m^3^)	0.027–0.030	*λ*_*V*_ = (*λ*_*L*_)^3^ = (1/50)^3^	(3.33–3.75) × 10^3^
*L* (m)	5.1	*λ*_*L*_ = 1/50	255
*H* (m)	3.5	*λ*_*L*_ = 1/50	175
*U* (m/s)	1.5–3.5	*λ*_*U*_ = (*λ*_*L*_)^1/2^ = (1/50)^1/2^	10.6–24.7
*H*_0_ (m)	0.19	*λ*_*L*_ = 1/50	9.5
*H*_e_ (m)	0.075	*λ*_*L*_ = 1/50	3.75
*D*_d_ (m)	0.04–0.08	*λ*_*D*_ = 1/20	0.8–1.6
*D*_s_ (m)	0.002–0.005	*λ*_*D*_ = 1/20	0.04–0.10

*λ*_*L*_ is the length scale ratio, respectively; *λ*_*M*_ and *λ*_*V*_ are the scale ratio of landslide mass and volume; *λ*_*D*_ is the scale ratio of debris avalanche’s particle diameter; *λ*_*U*_ is the scale ratio of flow velocity; *ρ*_d_ is the density of debris avalanche; *θ* is the terrain slope, which includes movement section (the former value) and accumulation section (the latter value); *g* is the gravity acceleration; *M*_d_ and *V*_d_ are the mass and volume of debris avalanche, respectively; *L* and *H* are the length and height in geometry dimension, respectively; *U* is the flow velocity; *H*_0_ and *H*_e_ are the initial thickness substrate and entrainment depth, respectively; *D*_d_ and *D*_s_ are the diameters of debris materials and substrate materials, respectively

Even if geometrical similarity and dynamically similarity are carefully designed for the physical modeling tests, it is impossible to uniformly replicate all dynamics in a flume system (Thompson and Wohl 1998). Thus, there still have some differences between the experiments results and corresponding actual situations in the landslide dynamics and entrainment effects, especially when the scale ratio is relatively small. However, the entrainment mechanisms behind the mass movement process are basically similar for the small scale of modeling test and large scale of actual landslide, which are discussed in detail in the following section.

### Entrainment mechanisms

**Fig. 10:**
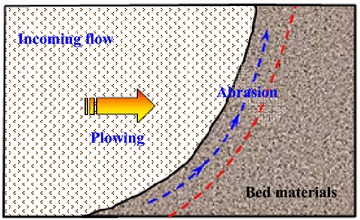
Sketch of the erosion mechanism observed in the experiments of Barbolini et al. (2005a)

The entrainment of the bed materials has long been considered as a critical phenomenon during the process of debris avalanches. Obviously, the research on the entrainment mechanism is of great importance to understanding the motion characteristics of debris avalanches, and many researchers have investigated it. For example, McDougall and Hungr (2005) considered that the failure of bed material during a landslide can be caused by the transmission of basal shear stress to the substrate, impacting and scouring, and generation of excess pore-water pressure because of rapid un-drained loading. Gauer and Issler (2004) hypothesized that the mechanisms of snow avalanches include impact erosion, abrasion, fluidization and plowing (as shown in Fig. 10). Barbolini et al. (2005b) observed two mechanisms through numerous laboratory tests, namely, plowing and abrasion. Dufresne (2012) considered that substrate entrainment mechanisms include plowing, fluidization or pore pressure changes when fluids are present, basal abrasion and deformation waves, which can accelerate the particles in the substrate bed.

**Fig. 11:**
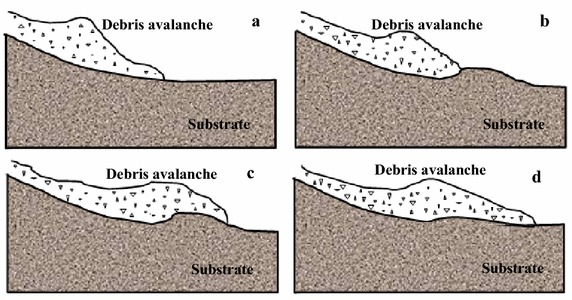
A hypothetical mechanism of momentum transfer between a moving rock mass and liquefiable substrate at full scale: **a** a rock mass approaching the substrate layer; **b** impact liquefaction, partial displacement and overriding of the substrate; **c** a mud wave projected forward; **d** a rock mass and mud wave deposit (modified from Hungr and Evans 2004)

In general, granular flows generally entrain substrate materials via impact erosion at the front of avalanches, plowing, basal abrasion, generation of excess pore-water pressures where fluids are present, and even fluidization (Gauer and Issler 2004; Dufresne 2012). Among these four mechanisms, plowing in the front of the avalanches is considered as the most efficient mechanism for substrate entrainment (Gauer and Issler 2004; Barbolini et al. 2005b; McDougall and Hungr 2005). Basal abrasion is observed to entrain much less bed materials than frontal entrainment of granular flows (Sovilla et al. 2006; Dufresne 2012) and is mainly responsible for the incorporation of eroded materials into the flow (Barbolini et al. 2005b). Although plowing is considered as the predominant mode of entrainment, each mechanism has the potential to entrain large amounts of bed materials under certain conditions (Gauer and Issler 2004). For example, substrate fluidization or the generation of excess pore-water pressures is rather important when the substrate is nearly saturated. Under this condition, Hungr and Evans (2004) considered that the rockslide front does not entrain the loose bed materials immediately, but overrides it partially and causes rapid loading and liquefaction of the saturated substrate. During this process, the avalanche exerts a great impact load on the substrate, causing liquefaction and rapid shearing of the bed materials, and thus entraining them, as seen in Fig. 11 (Hungr and Evans 2004).

**Fig. 12:**
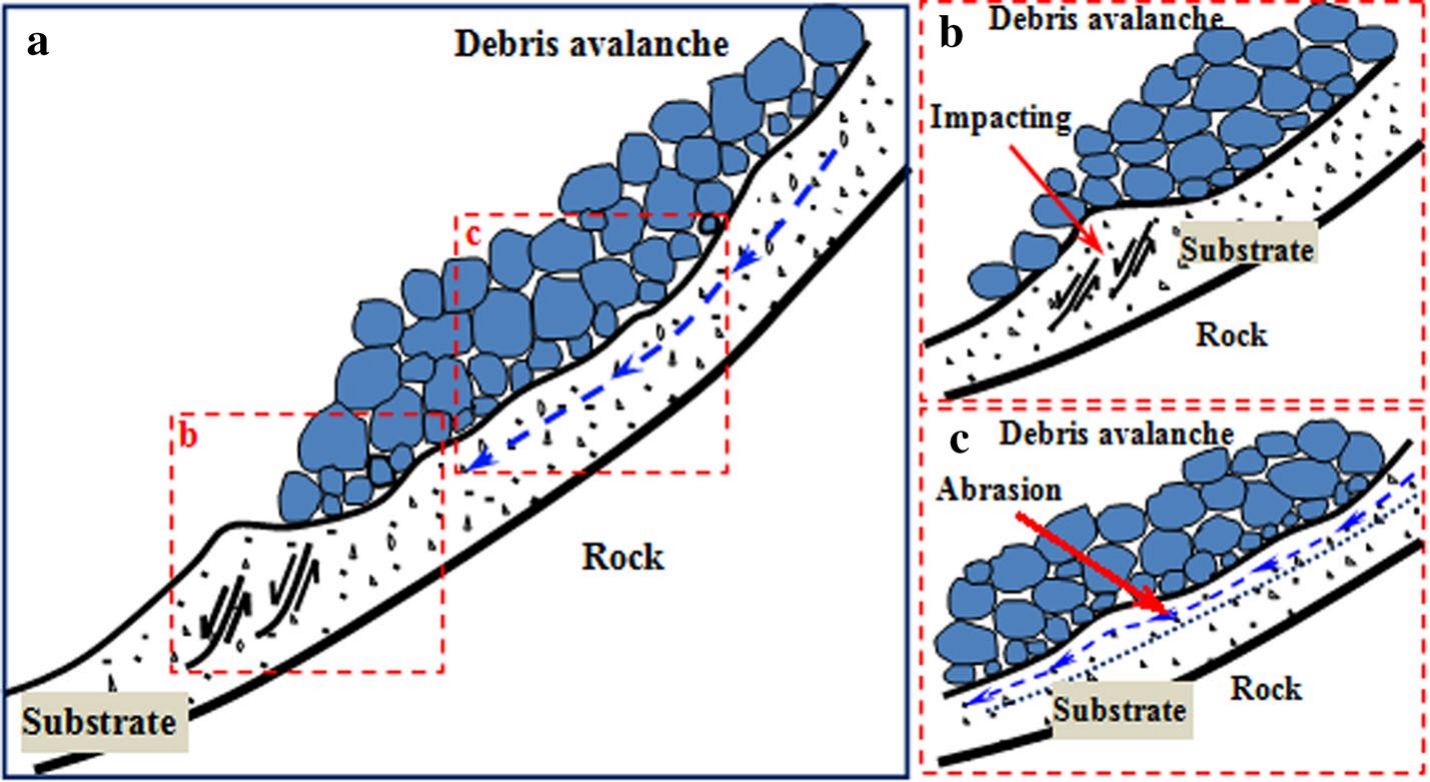
Mechanisms of the entrainment effect on bed materials during the mass movement process of a debris avalanche: **a** overview; **b** frontal impacting; **c** postmedian basal abrasion and erosion

According to the high-speed videos of our experiment, three main mechanisms can be observed from our experiment, including frontal impact erosion, plowing and basal abrasion in the postmedian of debris avalanches. A sketch of the entrainment mechanism can be seen in Fig. 12. Few researchers have considered impact erosion as a main factor in the entrainment process of debris avalanches. However, the paths of debris avalanche motion are inevitably irregular, and this will necessarily cause a continuous change of the moving direction of the avalanche materials. Granular flows will intensely collide with the substrate and erode it during the process.

**Fig. 13:**
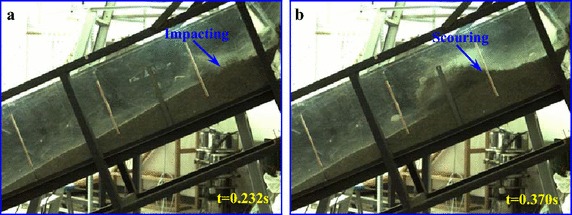
Impact erosion of the debris avalanche on loose substrate materials: **a** the frontal debris impact the static substrate; **b** the erosion process develops and the bed materials are intensively scoured

In our experiment, strong impact was observed as soon as the avalanche materials encounter the loose substrate materials, causing an intense spray of the bed materials (Fig. 13). The high-speed spray can cause a deadly catastrophe, e.g., the Frank landslide killed 73 people and buried numerous houses because of the high-speed spray of the Old Man River alluvial deposit (McDougall 2006). Strong impact can partly compact but also partly loosen the substrate entrained by the avalanche (Gauer and Issler 2004). Additionally, when the substrate contains water or even is saturated, under intense collision, the substrate will partly or completely liquefy under impact loading, which may greatly decrease the shear strength of the bed materials (Hungr and Evans 2004; Wang et al. 2003). Therefore, the previous impact can cause failure of the bed and contribute to the impact erosion of bed materials by the subsequent avalanche materials. According to Gauer and Issler (2004), the impact erosion speed increases with increasing particle size and impact angle, thus decreasing the inclination of the accumulation section or increasing the inclination of the movement section of the flume can enhance the effect of impact erosion.

**Fig. 14:**
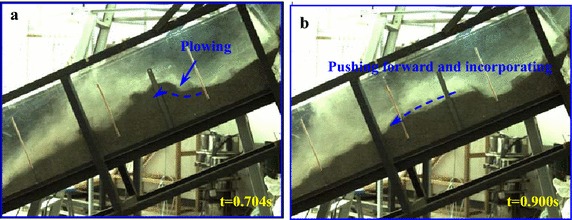
Plowing mechanism for the substrate entrainment: **a** the debris materials penetrate into the loose substrate and plow them; **b** the plowed deposits are pushed downward and finally incorporate into the avalanche

After the substrate is eroded for a certain depth, the bed materials will no longer spray, but rather be pushed forward, which is called plowing or ploughing as described above (Fig. 14). During this process, the substrate materials close to the impact area are plowed which causes thickening of the bed, part of the plowed materials are pushed forward by the front of the debris avalanche and part of them are incorporated into the flow body. According to the monitoring videos, this mechanism makes a great contribution to the entrainment of bed materials. Plowing generally is the dominant mechanism at the flow front, especially when there is a rapid decrease in the slope angle in the motion direction just like our experiment setup (McDougall and Hungr 2005). Therefore, decreasing the inclination change of the motion path of the debris avalanche can weaken the effect of plow for the because of penetrate depth.

**Fig. 15:**
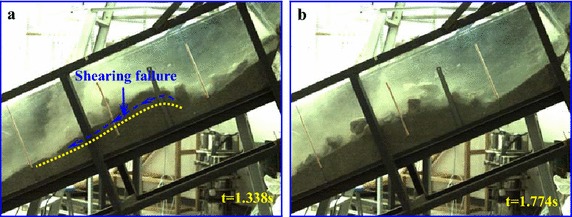
Basal abrasion of the substrate: **a** a thin layer of loose bed materials failures under the shear of debris avalanche; **b** the entrained materials flow forward and finally deposit

Another feature that can be seen from the videos is that a thin layer of bed materials flows downward under the shear strength of the debris avalanche (Fig. 15). This mechanism is called basal abrasion, which occurs when the avalanche materials slide parallel to the substrate materials, and hard particles plow grooves in the substrate under their own weight and the overburden load (Gauer and Issler 2004). The materials entrained under this mechanism are observed to have rate of a factor of 10 smaller than that in the front of the avalanche (Sovilla et al. 2006), and this mechanism predominantly occurs with high-strength substrate (Barbolini et al. 2005a; Dufresne 2012; Sovilla 2004). In fact, the substrate materials can be activated by the shear strength easier when the bed materials are relatively loose and have low resistance strength. However, under this condition, the avalanche will incorporate the substrate materials in the way of step entrainment which features that the avalanche slides on different layer of the bed along its length and entrains it (Sovilla 2004).

In nature, the volume of landslide–debris avalanches is generally huge and with high velocity, which means that they generally contain tremendous energy. Additionally, as is stated above, debris avalanches will continuously impact the substrate, thus causing a great failure of the substrate. When the substrate is loose, an intense impact can erode a rather large amount of bed materials directly, whereas when the substrate has a high density, the impact may only fragment, indirectly yielding bed materials. When the substrate fails from the impact of the following avalanche materials, it can be entrained if the shear strength of avalanche materials is larger than or equal to the resistance of the bed materials. Additionally, when the substrate is saturated, the great impact load may cause liquefaction, which can cause a decrease of resistance strength (Hungr and Evans 2004), which favors the process of basal abrasion.

Although the basal abrasion is considered much smaller than frontal entrainment by many researchers, it is rather apparent in our experiment and cannot be neglected. The thickness of the shearing moving layer is different along the accumulation section, namely, this layer is thicker near the impact zone. The reason for this phenomenon is that the depth and density as well as velocity of the debris avalanche are larger in the area close to the impact zone. As the flow moves down, it disperses and consumes energy, causing a thinner shearing layer. Our results conform to the theory of Hungr et al. (2005), which considers that the unstable depth of the substrate increases with the bulk density and thickness of the flows.

### Effect of avalanche mass and substrate thickness

The physical mechanical properties of substrate materials located along the landslide path, which can be characterized by different thicknesses, control the process of entrainment together with those of the moving mass (Crosta et al. 2013; Zhou et al. 2016b). In our experiment, the effect of substrate thickness and the mass (or volume) of the debris avalanche on substrate entrainment were studied. According the experiment results, increasing avalanche mass will enhance the entrainment effect due to greater entrainment energy and longer lasting of the erosion process. Many researchers have taken substrate thickness into account when studying the entrainment effect, e.g., Mangeney et al. (2007) found that the entrained substrate materials increase with increasing erodible bed thickness. Additionally, Hungr et al. (2005) also demonstrated that the amount of materials involved in the mass flow is limited to the depth of the weak erodible layer. Furthermore, Crosta et al. (2013) observed that there is no obvious erosion depth difference for different substrate thicknesses. However, in our study, both the erosion depth and the amount of entrained materials increased with decreasing substrate thickness. Our result can be explained by the following three aspects. First, according to Dufresne (2012), increasing basal boundary roughness of the loose substrate increases the stability of force chains and substrate resistance. Increasing substrate thickness leads to a growth of the normal stress on the base of the bed materials, increasing the resistance strength and stabilizing the force chain of the substrate materials. Additionally, the deformation when the mass impact with the bed of the thicker substrate will be more spread in the form of folding, which may consume more energy of the avalanche (Crosta et al. 2013). Furthermore, thicker substrate means a decrease in kinetic energy because of less accelerating time. Although erosion depth and the materials entrained by the debris avalanche increase with increasing substrate thickness in our study, substrate thickness will increase when the debris avalanche has sufficient energy, i.e., the avalanche is supply-limited (Mangeney et al. 2010).

**Fig. 16:**
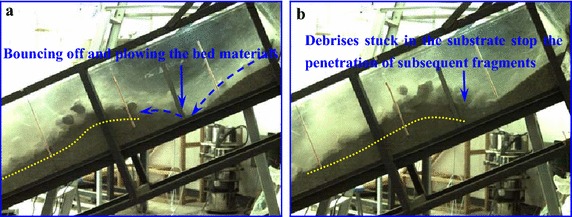
Effect of the substrate thickness on the entrainment process: **a** the maximum thickness of substrate is 13 cm; **b** the maximum thickness of substrate is 19 cm

In addition to influencing the amount of entrained bed materials, the variation of substrate thickness can also affect the style of substrate incorporation. As shown in Fig. 16, the effect of plowing weakens in the condition of thick substrate because the debris is easier to be stuck in the substrate which will stop the penetration of the subsequent rock fragments. Therefore, the substrate can only be pushed forward by the subsequent debris indirectly by the transmitting pressure. However, the bed materials tend to be directly plowed forward by the debris avalanche front with the decreasing thickness of substrate, which finally enhance the effect of plowing. Besides, the debris materials are easier to bounce off the flume rather than stuck on the substrate, which can cause greater motion energy and flow depth of the debris avalanche, strengthening basal abrasion. The above conclusions are drawn under the condition of our experiment. However, there will be different effect of the substrate thickness on the entrainment style when changing experiment condition, such as topography.

## Conclusions

Previous studies have indicated that the entrainment of substrate materials along the runout path of a debris avalanche can substantially increase the volume and change characteristics of the flow. The mechanisms of entrainment are very complex that laboratory tests were conducted herein and recorded by a high-speed camera. According to the monitoring videos, the substrate materials spray intensively as soon as they are impacted, and then are continually scoured by the debris avalanche. Under the drag of the debris avalanche, a thin layer of bed materials are activated to move, and the thickness of this layer decrease along the accumulation section. Therefore, based on these observations, conclusion can be drawn that the entrainment mechanisms of the debris avalanches in our experiments include impact erosion and basal abrasion. The impact erosion of bed materials is seen to be the predominant mode of entrainment. The amount of entrained materials and maximum erosion depth increases with increasing avalanche mass. However, the erosion depth increases nonlinearly with decreasing substrate thickness.
